# Deubiquitinating enzymes as oncotargets

**DOI:** 10.18632/oncotarget.3922

**Published:** 2015-04-23

**Authors:** Urszula L. McClurg, Craig N. Robson

**Affiliations:** ^1^ Solid Tumour Target Discovery Laboratory, Newcastle Cancer Centre, Northern Institute for Cancer Research, Medical School, Newcastle University, Newcastle upon Tyne, UK

**Keywords:** deubiquitination, DUBs, cancer, epigenetics, chromatin, androgen receptor, histones

## Abstract

Carcinogenesis is a complex process tightly regulated at multiple levels by post-translational modifications. Epigenetics plays a major role in cancer development, all stable changes to the gene expression process that are not a result of a direct change in the DNA code are described as epigenetics. Epigenetic processes are regulated by post-translational modifications including ubiquitination which can directly affect either histones or transcription factors or may target their co-factors and interacting partners exerting an indirect effect. Deubiquitination of these target proteins is equally important and alterations in this pathway can also lead to cancer development, progression and metastasis. Only the correct, unaltered balance between ubiquitination and deubiquitination ensures healthy cellular homeostasis. In this review we focus on the role of deubiquitinating (DUB) enzymes in various aspects of epigenetics including the regulation of transcription factors, histone modifications, DNA damage repair pathways and cell cycle regulation. We discuss the impact of those processes on tumourigenesis and potential therapeutic applications of DUBs for cancer treatment.

## INTRODUCTION

Ubiquitination is one of the most important post-translation modifications (PTMs) responsible for regulating the stability and activity of modified proteins. For the ubiquitin molecule to be attached to its target protein it initially has to be activated by an E1 enzyme during an ATP-dependent reaction, which is followed by conjugation of ubiquitin by an E2 class enzyme allowing E3 ubiquitin ligase to ubiquitinate target proteins directly or indirectly [1], [2], [3].

The consequences of ubiquitination depend on the type of chains formed during the process [4]. Proteins can be mono-, multi-mono- or poly-ubiquitinated. Ubiquitin contains seven lysine (K) residues with poly-ubiquitin chains linked through the K6, K11, K27, K29, K33, K48 and K63 residues. Mono-ubiquitination and K63 poly-ubiquitination have been linked to regulating protein activity [5]. K6, K11, K29 and K48 poly-ubiquitin chains control protein stability with K6 and K48 chains targeting proteins for proteosomal degradation, K11 is involved in endoplasmic reticulum mediated degradation pathways and control of cell cycle progression and K29 in regulating lysosomal degradation of proteins [6]. The role of K27 and K33 poly-ubiquitination is less understood however, they have been linked to innate immunity and immune responses.

### Deubiquitinating enzymes (DUBs)

Just as every action provokes a reaction all of the major post-translational modifications can be reversed. Enzymes that reverse PTMs are equally important to normal homeostasis as those that initially modify proteins. Protein ubiquitination by the E3 ligases can be reversed by deubiquitinating enzymes [7]. DUBs can be divided into five families; ubiquitin carboxy - terminal hydrolases (UCH), ubiquitin specific proteases (Usp), Otubain/Ovarian tumour - domain containing proteins (OTU), Machado - Joseph Domain (Josephin domain) - containing proteins (MJD) and Jab1/MPN domain associated metalloisopeptidase domain proteins. UCH, Usp, OUT and MJD proteins are all thiol proteases containing an active site cysteine which serves as a nucleophile facilitating the attack on lysine-glycine isopeptide bonds of ubiquitinated proteins. Jab1/MPN domain associated metalloisopeptidase domain proteins differ from the other DUB classes as they utilise a JAMM zinc metalloproteinase domain to break the bond between their target proteins and ubiquitin.

With increased appreciation for the critical importance of ubiquitination in cellular processes, DUBs role in health and disease is becoming a new focus of scientific research. Recent advances in the field uncovered a growing number of DUB substrates. DUBs control the stability and activity of multiple proteins crucial in cellular proliferation and survival including p53 - the guardian of the genome [8], [9], MDM2 [10], androgen receptor (AR) [11], [12], histones [13], [14], PHLPP and PHLPPL Akt phosphatases [15], [16], [17], Notch [18], NF-κB [19], [20], β-catenin [21], [22] and many more. It is now understood that a tight balance between ubiquitination and deubiquitination is required for cellular survival underlying the equal importance of E3s and DUBs.

### Epigenetics and DUBs in cancer

**Figure 1:**
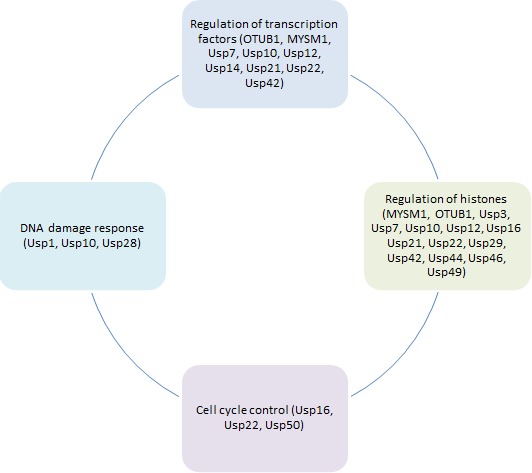
Role of deubiquitinating enzymes in different aspects of cancer epigenetic regulation

All stable, long-term alterations to the transcriptional potential of the cells that are not caused by direct changes to the DNA sequence itself and can be passed to daughter cells are referred to as epigenetics. These changes include DNA methylation, histone modifications and alterations to the activity of repressors and transcription factors. All of these processes result in altered transcriptome and affect cellular growth, survival and homeostasis; as such epigenetics play a crucial role in cancer development and progression [23]. DUBs can regulate all levels of epigenetic changes, by deubiquitinating proteins and changing their activity and / or stability DUBs control the levels of methylases and demethylases, histone proteins and their binding partners, repressors and transcription factors (Figure 1). DUBs can have both activating and repressing effects on gene transcription depending on their target proteins and the type of ubiquitin chain that is being removed. Additionally, DUBs can alter ubiquitin chains rather than remove them completely by for example deubiquitinating K48-linked chains and leaving the target protein in a mono-ubiquitinated state which results in altered protein fate. In this review we will focus on the role of DUBs in epigenetics and resulting cancer development. We will also review the potential therapeutic aspects of targeting DUBs to affect cellular epigenetics.

## REGULATION OF EPIGENETICS BY DEUBIQUITINATION

### Regulation of transcription factors

Gene expression is controlled by transcription factors that directly bind to DNA. Transcription factors act in complexes with other proteins and DUBs can affect their activity by deubiquitinating transcription factors directly or by targeting their binding partners and altering the stoichiometry of the complex. Usp12 activity towards the androgen receptor (AR) is an example of direct transcription factor deubiquitination [12]. AR is a member of the nuclear receptor superfamily and plays a key role in the transcriptional regulation of numerous genes important in the development of both normal and malignant prostate. AR deregulation is the key feature of prostate cancer (PC) development. Usp12 was reported to deubiquitinate the AR resulting in its increased protein stability and transcriptional activity [12]. As a result Usp12 promotes PC development with protein levels increased in PC patients compared to benign controls. However, AR has multiple ubiquitination sites and it is targeted by a variety of E3s, as a result AR deubiquitination doesn't always promote transcription. Usp26 can directly bind and deubiquitinate AR, acting as a co-regulator of the AR by reversing AR activation and degradation by MDM2 ubiquitination depending on cellular context highlighting the complexity of post-translational regulation [11]. Similarly, Estrogen Receptor (ER) can also be regulated by deubiquitination, OTUB1 has been identified to target ER-alpha affecting ER target gene transcription and stabilising its protein levels on chromatin [24].

Sometimes it is not clear if DUBs control transcription factors via direct deubiquitination or if their regulation of the transcriptome is more via effects exerted on histones that cause a signalling cascade affecting the transcription factors. This is the case for Usp10, it was initially reported to directly regulate p53 and AR by deubiquitinating them and acting as a co-activator [25]. However, more recent reports indicate that the effects on AR might be indirect via Usp10 activity towards mono-ubiquitinated histone H2A.Z [14]. As a result of Usp10's role in the regulation of histones and p53 it is frequently overexpressed in breast and brain cancer patients where its levels correlate with survival. In prostate cancer, with increasing grade of disease, the cellular localisation of Usp10 changes with expression becoming predominantly nuclear allowing for higher activity towards H2A.Z. Similarly MYSM-1 has been reported to indirectly regulate the AR. MYSM-1 can activate transcription of AR target genes via its involvement with p300 affecting histone acetylation and deubiquitination and binding of H1 to the nucleosome [26].

P53 is a crucial tumour suppressor acting as a transcription factor responsible for regulating the expression of multiple genes associated with stress responses, cellular survival, growth and homeostasis [27], [28], [29]. P53 can be directly deubiquitinated by Usp42 which reverses its ubiquitination by MDM2 [9]. Usp42 forms a direct complex with p53 and controls its activation in response to cellular stress; as a result it regulates p53-dependent transcription and cell cycle arrest. Usp7 is another DUB important for p53 activity [8]. Usp7 regulates the polycomb complex and factors associated with transcription including MDM2, p53 and FOXO. Usp7 directly deubiquitinates and stabilises p53, it is also necessary for p53 stabilisation by the tumour suppressor ING1 [30]. Additionally, independently of its deubiquitinase activity the DUB Usp7 regulates sequence specific RNA binding of the core domain of p53 thereby stimulating its transcriptional activity and expression of p21 [31]. Consequently, Usp7 inhibition was reported to inhibit cancer cell growth and increase apoptosis.

Some DUBs can control p53 stability independently of their enzymatic activity. One such example is OTUB1 which can inhibit UbcH5. UbcH5 is an MDM2 cognate ubiquitin-conjugating enzyme (E2), as a result of its inhibition p53 ubiquitination is abrogated leading to its increased stability and activity [32], [33]. Through binding to E2 enzyme Ubc13, OTUB1 also regulates the K63 ubiquitination of chromatin induced by DNA damage [34].

GATA3 serves as another example of a transcription factor regulated by deubiquitination. GATA3 is a master regulator of T helper cells (Th2) cell differentiation and function; it controls early T cell development and is one of only 3 genes mutated in over 10% of breast cancers as it plays a key role in mammary gland development [35], [36]. Recent reports demonstrate that GATA3 can be deubiquitinated by Usp21 which rescues it from proteosomal degradation and stabilises GATA3 protein levels [37]. This results in increased transcriptional activity of GATA3 and highlights the role of Usp21 in immune responses with Usp21 protein upregulated in regulatory T cells (T_regs_) from asthma patients, however the role of this interaction in carcinogenesis still remains to be established.

Both canonical and non-canonical Wnt signalling pathways are also regulated by deubiquitination. Recent reports identified proteasome associated DUB Usp14 as an oncogene and a positive regulator of Wnt signalling via deubiquitination of Dishevelled (Dvl) [38]. Depletion of Usp14 attenuated downstream Wnt signalling which was further evidenced when correlation between the levels of Usp14 and β-catenin in colon tissues was observed. Usp14 was also up regulated in non-small cell lung carcinoma where high levels correlated with decreased survival and poor prognosis [39]. This was attributed to the oncogenic properties of Usp14 with silencing causing cell cycle arrest as a consequence of β-catenin degradation.

An additional level of control of transcriptional activity mediated by the nuclear receptors is provided by the SAGA (Spt-Ada-Gcn5-acetyl 1 transferase) histone acetyltransferase (HAT) complex. In addition to HAT activity, SAGA has deubiquitinating activity, which is required for the transcriptional activity of nuclear receptors with Usp22 being the active DUB subunit of SAGA that removes ubiquitin from the histones H2A and H2B [40]. As SAGA is a chromatin modifying transcription coactivator complex which regulates the expression of genes related to tumourigenicity and proliferation its levels predict treatment failure and are also used as a marker of recurrence, metastasis and resistance to therapy with levels significantly increased in cancers, including colorectal cancer [41], [42].

### Regulation of histones

Mono-ubiquitination of histone H2A is one of the key histone modifications, it is associated with transcriptional repression by the polycomb group proteins and maintenance of the genome integrity. H2A is mono-ubiquitinated at K119 and it is estimated that between five and fifteen percent of the H2A fraction is mono-ubiquitinated at any one time compared to only one percent of H2B. As previously discussed, Usp10 can deubiqutinate the mono-ubiquitinated H2A.Z variant of H2A which results in increased AR activity [14]. However, H2A can also be deubiquitinated by MYSM1 (2A-DUB) [26] and UbpM (Usp16) in a way that is independent of its phosphorylation by CDK1 [43], [44]. UbpM specifically deubiquitinates histone H2A, but not H2B. This deubiquitination is required for dephosphorylation of histone H3 on S10 resulting in chromosome segregation during the mitotic entry. As a result, UbpM silencing decreases cell growth due to defects in mitosis. UbpM similarly controls Hox gene expression via regulation of H2A ubiquitination status. Ubiquitination of H2A is known to contribute to the embryonic stem cell (ESC) pluripotency by repressing lineage-specific gene expression [45]. It has been reported that deubiquitination of H2A by UbpM controls gene expression in ESCs with UbpM binding to the promoter regions of multiple genes in ESCs and regulating the H2A ubiquitination levels. It is now established that UbpM is required for ESCs to differentiate, as in its absence ubiquitinated H2A mediates repression of lineage-specific genes expression abrogating cellular differentiation [45].

Histone ubiquitination plays a vital role in both DNA damage response and repair pathways (Table 1). Increased mono-ubiquitination of H2A, H2B, H3 and H4 has been previously observed upon DNA damage. During the early DNA damage response, DNA-PK, ATM and ATR kinases phosphorylate a fraction of the H2AX variant of H2A, which is commonly referred to as γH2AX [46], [47]. RNF168 is an E3 ligase that ubiquitinates histones H2A and γH2AX during the DNA damage response [48], this ubiquitination can be reversed by Usp3 [49]. Usp3 associates with chromatin and deubiquitinates H2A at K13 and K15 and γH2AX at K118 and K119. As a result Usp3 overexpression has been shown to impair the accumulation of the BRCA1 and 53BP1 repair factors at the DNA damage sites in response to DNA damage and to counteract the activity of RNF168. Consistent with these findings, Usp3 ablation caused accumulation of DNA breaks and activation of DNA damage checkpoint pathways. This pathway can also be reversed by other DUBs, including Usp21 [50], OTUB1, Usp29 and Usp44 [51]. Both Usp44 and Usp29 deubiquitinate H2A with USP44 being recruited to the RNF168-generated ubiquitination products at double stranded break sites [51].

Ubiquitination of both H2A and H2B is equally important for maintaining cellular homeostasis and there are DUBs that specifically target histone H2B, including Usp49 [52] and Usp42 [53]. H2B ubiquitination regulates H3K4 and H3K79 methylation and impacts on the chromatin structure [52]. Usp49 in complex with RVB1 and SUG1 yeast homologues deubiquitinates H2B, this modification is required for efficient co-transcriptional splicing of a large set of exons. Silencing Usp49 induces relatively small changes in gene expression, however alterations in H2B ubiquitination levels caused by Usp49 regulate U1A and U2B association with chromatin and binding to nascent pre-mRNA. Consequently, Usp49 plays a crucial role in co-transcriptional pre-mRNA processing [52]. H2B ubiquitination can additionally be reversed by Usp7 resulting in epigenetic silencing of homeotic genes [54].

Some DUBs are not as specific and have the ability to deubiquitinate both histones H2A and H2B, for example the closely related family of DUBs comprising Usp1, Usp12 and Usp46. All three DUBs require Uaf-1 (WDR48) for their enzymatic activity [55] and additionally Usp12 and Usp46, but not Usp1, activity is further enhanced by binding to WDR20 [56]. Usp12 and Usp46 deubiquitinate both H2A and H2B and Uaf-1 is required for this reaction [13]. Usp22 can also reverse the polycomb complex mediated ubiquitination of H2A and H2B causing multiple changes in gene expression profiles including transcriptional activation of MDM2 and Hox [57], [58]. Recent reports however, indicate that even though Usp22 is active towards both H2A and H2B it preferentially targets H2B for which it is one of the main DUBs.

### Cell cycle regulation and DNA damage response

In normal cells most genes have an epigenetically stable transcriptional status. However, some genes are an exception to this rule as their sole purpose is to be responsive to the outside stimuli including growth factors and cellular contact. Those genes are most susceptible to epigenetic changes and their expression is rapidly affected by them. Dysregulation of epigenetics might result in altered expression of these genes leading to cellular transformation and malignancy. As a result, impact of DUBs on cell cycle regulation and DNA damage repair pathways deserves specific attention. The human genome is continuously challenged by both endogenous and exogenous insults potentially damaging the DNA which can result in various types of damage including double and single strand breaks, oxidative lesions and pyrimidine dimers. Cells have developed multiple ways to counteract and repair the DNA damage known as the DNA damage response pathways. These responses can be divided into two main groups; cell-cycle checkpoint activation and DNA repair. Both of these mechanisms are tightly controlled by chromatin remodelling and epigenetics.

As discussed in previous sections multiple DUBs affect cell cycle indirectly by regulating transcription factors that control cell cycle progression, including the AR and p53, and by modulating histones. However, some DUBs regulate cell cycle in a much more direct fashion. DUBs can regulate cell cycle progression by controlling the G2/M checkpoint. Specifically both Usp50 and UbpM contribute towards this process. Usp50, even though it is catalytically inactive, plays a role in cell cycle progression. It associates with Hsp90 and controls Wee1 stability via an Hsp90-dependent mechanism. Usp50 consequently functions as a negative regulator of the G2/M checkpoint [59]. UbpM regulates the same checkpoint but via a different mechanism. Following S552 phosphorylation UbpM translocates to the nucleus and regulates the cell cycle G2/M phase progression and cell proliferation [44]. Additionally, Usp22 directly deubiquitinates TRF1 (TBP(TATA box-binding protein)-related factor 1) to regulate the transcription of cell cycle and apoptosis genes [60] and inhibits the transcriptional activity of p53 by deubiquitinating SIRT1 histone deacetylase [61] and by regulating MDMX stability [62].

Usp1 is a key protein involved in the DNA damage response. Indeed inhibition of the Usp1-Uaf-1 complex sensitises cells to chemotherapy. Usp1 counteracts the mono-ubiquitination of PCNA which prevents recruitment of low fidelity DNA polymerases in the absence of DNA damage [63]. It is also involved in double strand DNA break repair through the homologous recombination pathway. Additionally, Usp1 deubiquitinates and stabilises ID (Inhibitor of DNA binding) proteins 1, 2 and 3, as ID can inhibit differentiation this preserves the undifferentiated state of cells [64]. Usp1 modulates DNA replication, polymerase choice and DNA repair by PCNA and as a result *Usp1* knock-out mice are genetically unstable and hypersensitive to DNA damage [65], [66].

Usp10 is involved in DNA damage response control via regulation of the p53 protein. Upon DNA damage, Usp10 is phosphorylated which is essential for Usp10 to relocate to the nucleus, allowing it to stabilise p53 [67]. The DNA damage response is also regulated by the Chk2-p53-PUMA pathway in response to double strand breaks *in vivo*. This process is tightly controlled by Usp28 which is necessary to stabilise Chk2 and 53BP1 in response to DNA damage and is required for DNA damage induced apoptosis [68]. Usp28 is found to be recruited to double strand breaks and this is dependent on the 53BP1 protein.

### Regulation of deubiquitinating enzyme activity

Activity and stability of multiple DUBs is regulated by posttranslational modifications and interactions with other proteins. Commonly enzymes are regulated by phosphorylation, this is also frequent amongst DUBs. To allow its activity towards p53, the Usp10 protein needs to be phosphorylated at T42 and S337 by ATM, this occurs as a result of DNA damage and stabilises Usp10 allowing it to translocate to the nucleus and deubiquitinate p53 [67]. Interestingly in prostate cancer with increased cancer grade and metastasis the expression of Usp10 becomes predominantly nuclear which could possibly be associated with elevated DNA damage. Phosphorylation of Usp10 by ATM is also required for Usp10 antioxidant activity in stress granules [69]. Phosphorylation of UbpM at Ser552 by cyclin-dependent kinase 1 (CDK1) is also needed for its translocation to the nucleus and regulation of the cell cycle G2/M phase progression and cell proliferation but, this phosphorylation is not required for its deubiquitinase activity, substrate specificity and regulation of gene expression [44]. Similarly, Usp7 short isoform (Usp7S) can be phosphorylated by CK2 at S18 leading to its protein stabilisation which subsequently increases the pool of MDM2 and decreases the levels of p53 protein [70]. This is reversed upon irradiation by an ATM dependant phosphatase PPM1G [70]. DUBs can play a role in infection and immunity and their activity can be regulated by bacterial kinases. During *Yersinia* infection OTUB1 can be phosphorylated by a bacterial kinase YpkA which modulates cellular susceptibility to *Yersinia* invasion [71].

Many of DUB phosphorylation sites are highly conserved throughout evolution. Yeast homologue of Usp12 (Ubp9) has been shown to be phosphorylated and this phosphorylation was lost upon deletion of both of its co-factors Uaf-1 and WDR20 highlighting its potential role in enzymatic activity [72]. Similarly close family member of Usp12, Usp1 is also phosphorylated at S313 by CDK1 during mitosis, this modification is required for its interaction with Uaf-1 as it lies within the Uaf-1 binding region (amino acids 235-408). Consequently, it is also a pre-requisite for Usp1 DUB activity as complex formation with Uaf-1 is required [73], [74]. Conversely phosphorylation at S42 and S67 have not been attributed any physiological roles to date [73]. S313 of Usp1 lies within both the consensus sequence for CDKs and also Usp1s region 307-330 responsible for APC/C cdh1 mediated Usp1 degradation during G1 phase. Consequently S313 phosphorylation plays a crucial role in maintaining protein stability during mitosis as Usp1 is ubiquitinated during G1 by APC/C cdh1 leading to its proteosomal degradation. Usp1 can be additionally stabilised by CAPNS1 which activates Cdk5 resulting in inhibition of cdh1 subsequently inhibiting Usp1 degradation [75].

Frequently the activity of deubiquitinating enzymes is regulated by interactions with various binding partners. TRAF2 can bind to Usp2a which inhibits its effect on K48 but not K63 linked poly-ubiquitin chains, consequently the ratio between TRAF2 and Usp2a determines cells sensitivity to cell death [76]. Usp10, alongside Usp13, is regulated by binding to Beclin-1 which affects their protein stability, activity and subsequent deubiquitination of target proteins [77]. Usp22 activity is regulated by histone deacetylases, their inhibition abrogates the binding of RNA Polymerase II to Usp22 promoter supressing its transcription [78].

DUB protein stability has a crucial role in regulating enzymatic activity; some DUBs can auto-regulate themselves others are ubiquitinated/SUMOylated. For example, Usp7 is activated by its own *C*-terminal domain [79]. Conversely, Usp1 has the ability to auto-cleave itself at a di-glycine motif leading to its degradation [80]. OTUB1 is regulated by mono-ubiquitination at K59 and K109 which is required for it to inhibit the E2 UhcH5 resulting in MDM2 inhibition and p53 activation [33]. Binding of OTUB1 and E2 enzymes, including UbcH5 and Ubc13, also regulates OTUB1 enzymatic activity towards K48 ubiquitin chains [81]. Usp28 on the other hand can be regulated by SUMOylation at the *N*-terminal domain, this has a negative effect on its deubiquitinase activity [82].

Splicing can also play a role in the regulation of DUBs activity and cellular localisation. Both Usp7 and Usp21 have been reported to undergo alternative splicing. Usp21 short variant lacks the nuclear export sequence (NES) and as a result localises predominantly in the nucleus however, that has no major effect on its enzymatic activity as both full length and variant Usp21 affect H2A ubiquitination to a comparable degree *in vitro* but variant was observed to be more active *in vivo* due to its localisation [83].

**Table 1:** Role of DUBs in histone deubiquitination and the cellular consequences

DUB	DUB Family	Histone substrates	Cellular processes
Usp3	Usp	H2A and H2B	DNA repair and cell cycle progression
Usp7	Usp	H2A and H2B	Gene expression, protein stability, cell cycle progression and proliferation
Usp10	Usp	H2A	DNA repair and transcription
Usp12	Usp	H2A and H2B	Transcription
UbpM (Usp16)	Usp	H2A	DNA repair, cell cycle progression, differentiation and proliferation
Usp21	Usp	H2A	Gene expression and DNA repair
Usp22	Usp	H2A and H2B	Gene expression, protein stability, proliferation and cell cycle progression
Usp29	Usp	H2A	DNA repair and transcription
Usp44	Usp	H2A	DNA repair, cell cycle progression and differentiation
Usp46	Usp	H2A and H2B	Transcription
Usp49	Usp	H2B	Gene expression and pre-mRNA processing
MYSM1	JAMM	H2A	Transcription, haematopoiesis
OTUB1	OTU	H2A	DNA repair

### Targeting DUBs

DUBs are key enzymes which regulate cellular growth, survival and homeostasis through multiple pathways including epigenetics, as such aberrations in DUB signalling and activity can play crucial roles in cancer development, progression and metastasis. This is evidenced by multiple carcinogenic agents that exert their effects via the DUB pathway. Carcinogenic properties of nickel compounds have been attributed to the increased ubiquitination of H2A and H2B [84]. However, nickel compounds do not affect histone ubiquitination directly but rather inhibit the DUBs responsible for reversing this process, as such increasing the pool of ubiquitinated histones. As a consequence, targeting DUBs might prove to be a valid strategy for developing novel anti-cancer therapeutics (Table 2).

**Table 2:** Currently available agents aimed at DUBs discussed in this review

DUB	Compound	Other targeted DUBs	Reference
Usp1	Pimozide ML323 SJB2-043 and SJB3-019A GW7647	Usp2, Usp5, Usp7, Usp8, Usp46 - - -	[87] [88], [89], [90] [91] [87]
Usp7	HBX 19,818 and HBX 28,258 P5091 and P22077 Pimozide	Usp47 - Usp1, Usp2, Usp5, Usp8, Usp46	[93] [95], [96], [97] [87]
Usp10	Spautin-1	Usp13	[77]
Usp14	b-AP15 AC17	UCHL5 UCHL5	[98] [99]
Usp46	Pimozide	Usp1, Usp2, Usp5, Usp7, Usp8	[87]

Targeting DUBs as an anti-tumourigenic therapeutic strategy has its proof of principle in the use of bortezomib, a broad range inhibitor of the ubiquitin proteasome system, in multiple melanoma treatment [85]. This strategy is however limited by the lack of specificity of bortezomib which results in toxicity. Targeting individual DUBs that play a role in particular cancers is predicted to be a much better strategy. However, targeting single DUBs is a very complex challenge due to the high levels of homology, particularly between the catalytically active domains, and promiscuity. Most DUBs target multiple proteins and additionally many of the DUB targets can be deubiquitinated by more than one DUB. The same DUB can target proteins from the same pathway that exert opposing effects, for example Usp7 can deubiquitinate both p53 and its E3 ligase MDM2 depending on the circumstances. In this context broad spectrum inhibitors targeting closely related families of DUBs that deubiquitinate the same substrates might in fact be a valuable strategy.

As mentioned previously multiple DUBs rely on co-factors, such as WD40 proteins, for their activity (Table 3), [86], [7]. This poses a very exciting drug design opportunity and offers a chance for developing much more specific agents when the binding of two proteins rather than a conserved active domain of one of them is being targeted. As previously discussed, Usp1 is responsible for DNA damage response pathway regulation and its activity is dependent on binding to its cofactor, Uaf-1. This allowed for the identification of more specific agents targeting the Usp1 protein's interaction with Uaf-1 rather than Usp1 directly, thus circumventing the potential issue of sequence homology and conserved domains within similar DUBs. Three Usp1 inhibitors explored this strategy, Pimozide [87], ML323 [88], [89], [90] and GW7647 [87] each targeting the Usp1-Uaf-1 complex in a non-competitive manner. Usp1 known targets include the Fanconi anaemia complex proteins, FANC1 and FANCD2, PCNA and the inhibitor of DNA binding (ID) transcription factors [87]. As *Usp1*-null mice have been shown to be hypersensitive to DNA damage it is likely that targeting Usp1 could increase the sensitivity of cancer cells to DNA damaging agents [66]. Multiple Usp1 inhibitors have been identified via both compound library screening and drug development programs. Therapeutic inhibition of Usp1 was previously reported to re-sensitise cisplatin-resistant non-small-cell lung cancer cells to the drug [87]. This result was confirmed by an observed increase in the mono-ubiquitination of PCNA and FANCD2 upon Pimozide treatment which indicated successful targeting of Usp1. Later reports supported this observation with the ML323 compound decreasing the cells ability to repair the DNA damage and potentiating the effects of cisplatin [88]. The authors proposed synthetic lethality as an explanation for this observation; because the DNA damage repair pathway is inhibited upon Usp1 targeting then damage induced by cisplatin is not being repaired by the cells, consequently resulting in cell death. More recently developed compounds SJB2-043 and SJB3-019A inhibited the salvaging of ID1, ID2, ID3, FANC1 and FANCD2 proteins by Usp1 from proteosomal degradation and as a result caused an increase in cell death and sensitisation to DNA damaging agents [91].

Usp7 is one of the most extensively researched DUBs due to its role in regulating multiple key proteins including p53, MDM2, PTEN, FOXO, the polycomb complex and histones [92]. During cancer development, Usp7 plays an oncogenic role as it promotes cellular survival. Most recent attempts at targeting Usp7 include development of P5091, HBX 19,818 and HBX 28,258 inhibitors [93]. All of these compounds were shown to abrogate the effects of Usp7 on p53, they also inhibit the enhanced tumourigenicity of claspin caused by Usp7 activity and increase apoptosis in cancer cells [94]. Promising data arose from the application of P5091 and its second-generation derivative P22077 in an investigation where both compounds were capable of increasing survival when used as single agents in a xenograft study. Additionally, when combined with standard treatments synergistic effects and re-sensitisation were observed for both compounds in multiple melanoma [95] and neuroblastoma [96], respectively. These results are very promising as resistance is one of the major challenges of present cancer management. As such, developing compounds focussed upstream of the present therapeutic targets could help to both avoid developing future resistance and also allow to re-sensitise patients resistant to the available DNA damaging agents by exploring synthetic lethality and combining them with inhibitors of DUBs responsible for DNA damage repair.

**Table 3:** List of DUBs discussed in this review that rely on binding to WD40 proteins

DUB	WD40	Cellular processes
Usp1	WDR48 (Uaf-1)	DNA damage response, Fanconi anaemia pathway, homologous recombination, cellular differentiation and Akt signalling
Usp3	WDTC1	DNA repair and cell cycle progression
Usp7	BUB3, WDR21A, RAE1	Gene expression, protein stability, cell cycle progression and proliferation
Usp12	WDR20, WDR26, WDR48, WDR77, DMWD	Transcription, Notch signalling and Akt signalling
Usp22	TAF5L	Gene expression, protein stability, proliferation and cell cycle progression
Usp42	WDR18	Transcription, stress response and cell-cycle progression. When fused to RUNX1 it is involved in pathogenesis of acute leukaemia
Usp44	TBL2	DNA repair, cell cycle progression and differentiation
Usp46	WDR20, WDR26, WDR48, WDR77, DMWD	Transcription, nervous system development and Akt signalling
Usp49	COPA	Gene expression and pre-mRNA processing
Usp50	PRPF4	Cell cycle progression

## CONCLUSIONS AND FUTURE PROSPECTS

Discoveries over recent years clearly indicate that ubiquitination and deubiquitination regulate cellular homeostasis and as a result deregulation of these processes can promote cancer development and progression. DUBs can affect carcinogenesis through multiple cellular pathways with epigenetics being the main example. Deubiquitination of histones, transcription factors and their co-factors plays a major role in regulating epigenetics by DUBs. As a result DUBs could be valuable therapeutic targets in oncology. Although this field is still relatively novel, recent advances indicate that targeting DUBs could be an efficient strategy. Novel inhibitors aimed at both Usp1 and Usp7 have been shown to re-sensitise cells to known therapeutic agents and to have a therapeutic effect when used independently in both the cellular setting and in some xenograft models. DUBs might present a challenge for compound design as their active domains are often conserved and many of them are Cys-dependent making targeting more complex. Recently, Usp1 inhibitors have been discovered which inhibit Usp1 by targeting its interaction with Uaf-1, a protein binding partner required for Usp1's enzymatic activity, rather than by targeting the active domain directly. This is a very exciting direction which could offer a solution to DUB inhibitors design providing both specificity and decreased toxicity. Multiple DUBs rely on binding to other proteins for their activity and stability and many of those interacting partners are WD40 proteins just like Uaf-1 (Table 3), it does however remain to be established if this strategy will prove therapeutically advantageous.
